# Mutations in *CDK5RAP2* cause Seckel syndrome

**DOI:** 10.1002/mgg3.158

**Published:** 2015-05-24

**Authors:** Gökhan Yigit, Karen E Brown, Hülya Kayserili, Esther Pohl, Almuth Caliebe, Diana Zahnleiter, Elisabeth Rosser, Nina Bögershausen, Zehra Oya Uyguner, Umut Altunoglu, Gudrun Nürnberg, Peter Nürnberg, Anita Rauch, Yun Li, Christian Thomas Thiel, Bernd Wollnik

**Affiliations:** 1 Institute of Human Genetics, University of CologneCologne, Germany; 2 Center for Molecular Medicine Cologne (CMMC), University of CologneCologne, Germany; 3 Cologne Excellence Cluster on Cellular Stress Responses in Aging-Associated Diseases (CECAD), University of CologneCologne, Germany; 4 Chromosome Biology Group, MRC Clinical Sciences Centre, Imperial College School of Medicine, Hammersmith HospitalLondon, W12 0NN, UK; 5 Department of Medical Genetics, Istanbul Medical Faculty, Istanbul UniversityIstanbul, Turkey; 6 Institute of Human Genetics, Christian-Albrechts-University of KielKiel, Germany; 7 Institute of Human Genetics, Friedrich-Alexander University Erlangen-NurembergErlangen, Germany; 8 Department of Clinical Genetics, Great Ormond Street Hospital for ChildrenLondon, WC1N 3EH, UK; 9 Cologne Center for Genomics, University of CologneCologne, Germany; 10 Institute of Medical Genetics, University of ZurichSchwerzenbach-Zurich, Switzerland

**Keywords:** *CDK5RAP2*, *CEP215*, microcephaly, primordial dwarfism, Seckel syndrome

## Abstract

Seckel syndrome is a heterogeneous, autosomal recessive disorder marked by prenatal proportionate short stature, severe microcephaly, intellectual disability, and characteristic facial features. Here, we describe the novel homozygous splice-site mutations c.383+1G>C and c.4005-9A>G in *CDK5RAP2* in two consanguineous families with Seckel syndrome. *CDK5RAP2* (*CEP215)* encodes a centrosomal protein which is known to be essential for centrosomal cohesion and proper spindle formation and has been shown to be causally involved in autosomal recessive primary microcephaly. We establish *CDK5RAP2* as a disease-causing gene for Seckel syndrome and show that loss of functional CDK5RAP2 leads to severe defects in mitosis and spindle organization, resulting in cells with abnormal nuclei and centrosomal pattern, which underlines the important role of centrosomal and mitotic proteins in the pathogenesis of the disease. Additionally, we present an intriguing case of possible digenic inheritance in Seckel syndrome: A severely affected child of nonconsanguineous German parents was found to carry heterozygous mutations in *CDK5RAP2* and *CEP152*. This finding points toward a potential additive genetic effect of mutations in *CDK5RAP2* and *CEP152*.

## Introduction

Seckel syndrome (MIM 210600 for SCKL1) is an autosomal recessive disorder characterized by intrauterine growth retardation, proportionate short stature, severe microcephaly, intellectual disability, and a typical facial appearance with a prominent and beaked nose, sloping forehead, and micrognathia (Seckel 1960; Majewski et al. 1982a). Most often, intellectual disability is associated with variable structural brain anomalies, and this combination mainly distinguishes Seckel syndrome from an overlapping primordial dwarfism syndrome known as microcephalic osteodysplastic primordial dwarfism type II (MOPD II, MIM 210720) (Hall et al. 2004; Willems et al. 2010).

Seckel syndrome is a genetically heterogeneous disorder; autosomal recessive mutations in eight different genes have been identified to date. They include very rare mutations in the ataxia-telangiectasia and Rad3-related (*ATR*, MIM 601215) gene, encoding a phosphatidylinositol 3-kinase-like kinase with distinct functions in DNA damage response, and mutations in *ATRIP* (MIM 606605), *CENPJ* (MIM 609279), *RBBP8* (MIM 604124), *NIN* (MIM 608684), *DNA2* (MIM 601810), *CEP63* (MIM 614724), and *CEP152* (MIM 613529) (Borglum et al. 2001; O’Driscoll et al. 2003; Al-Dosari et al. 2010; Kalay et al. 2011; Sir et al. 2011; Dauber et al. 2012; Ogi et al. 2012; Qvist et al. 2012; Shaheen et al. 2014). Mutations are most frequently found in *CEP152*, a centrosomal protein with essential roles during mitosis and in DNA damage response (Kalay et al. 2011). In this study, we present homozygous causative mutations in *CDK5RAP2* (MIM 608201, NM_018249.5, NP_060719) in two families with Seckel syndrome and demonstrate that severe defects in mitosis and spindle organization underlie the molecular pathogenesis of the disease. In addition, we report a patient with Seckel syndrome likely caused by digenic inheritance of heterozygous mutations in *CDK5RAP2* and *CEP152*.

## Materials and Methods

### Subjects

The study was performed in accordance with the Declaration of Helsinki protocols. We collected peripheral blood samples from the affected children and parents after informed consent was obtained according to the protocols approved by the ethics committees of the participating institutions. Written consent for publication of the photographs was given. DNA from participating family members was extracted from peripheral blood lymphocytes by standard extraction procedures.

### Linkage analysis

We performed genome-wide linkage analysis in two families (SK-1 and SK-2) using the Affymetrix GeneChip® Human Mapping 250K Sty Array (Affymetrix, Santa Clara, CA). We verified sample genders by counting heterozygous SNPs on the X chromosome. Relationship errors were evaluated with the help of the program Graphical Relationship Representation (Abecasis et al. 2001). The program PedCheck was applied to detect Mendelian errors (O’Connell and Weeks 1998) and data for SNPs with such errors were removed from the data set. Non-Mendelian errors were identified by using the program MERLIN (Abecasis et al. 2002) and unlikely genotypes for related samples were deleted. Linkage analysis was performed assuming autosomal recessive inheritance, full penetrance and a disease gene frequency of 0.0001. Multipoint LOD scores were calculated using the program ALLEGRO (Gudbjartsson et al. 2000). Haplotypes were reconstructed with ALLEGRO and presented graphically with HaploPainter (Thiele and Nürnberg 2005). All data handling was performed using the graphical user interface ALOHOMORA (Ruschendorf and Nürnberg 2005).

### Mutation screening

We identified candidate genes in the critical region using the ENSEMBL (http://www.ensembl.org) and UCSC (http://www.genome.ucsc.edu) human genome databases. All exons and intron-exon boundaries of the *CDK5RAP2* gene were amplified from DNA of index patients from all families and we sequenced the PCR products by BigDye Terminator method on an ABI 3100 sequencer. Identified mutations were resequenced in independent experiments and tested for cosegregation within the families. 150 healthy control individuals from Turkey and 282 controls from Pakistan were screened for each mutation by PCR.

### Cell lines and cell cultures

HEK293T cells and primary fibroblast cell lines established from patient SK-1 II.1 were cultured in Dulbecco’s modified Eagle medium (DMEM; Gibco, Life Technologies, CA, Carlsbad) supplemented with 10% fetal calf serum (FCS; Gibco) and antibiotics. For H2AX activation, cells were either treated for 1 h with 1 mmol/L Hydroxyurea (HU; Sigma-Aldrich, St. Louis, MO) or irradiated with 10 J/m^2^ UV-C, incubated for 24 h and then subjected to Western blot analysis. MG-132 (Sigma-Aldrich) was used with concentrations of 10 *μ*mol/L for the indicated times.

### Minigene assay/pSPL3 splicing assay

In vitro analysis of the potential splice-site mutation c.4005-9A>G was performed using the pSPL3 splicing assay. Fragments of the human *CDK5RAP2* gene containing exon 27, flanked by 600 bp of upstream intronic sequence and 700 bp of downstream intronic sequence, were cloned into the splicing vector pSPL3. The previously described splice-site mutation c.4005-15A>G in *CDK5RAP2* was introduced via site-directed mutagenesis (Bond et al. 2005). Plasmids were transfected into HEK293T cells and mRNA was isolated and reverse transcribed as described below.

### cDNA analysis

RNA was extracted from HEK293T cells and primary fibroblasts using the RNeasy® Mini Kit (Qiagen, Hilden, Germany). One microgram of total RNA was reverse transcribed using the RevertAid™ First Strand cDNA Synthesis Kit (Fermentas, St. Leon-Rot, Germany) and RT-PCR products were used for *CDK5RAP2*-specific PCR amplification.

### Protein isolation and analysis

All cells were solubilized using ice-cold RIPA buffer (10 mmol/L Tris, pH: 8.0; 150 mmol/L NaCl; 1 mmol/L Ethylendiamintetraacetate (EDTA); 10 mmol/L NaF; 1 mmol/L Na_3_VO_4_; 10 *μ*M Na_2_MoO_4_; 1% NP-40; protease inhibitors P 2714 [Sigma-Aldrich]) and total protein concentration of extracts was determined using the BCA Protein Assay Kit (Thermo Fisher Scientific, Life Technologies, Carlsbad, CA). Twenty-five micrograms of total protein from each sample was separated by 4–12% SDS-PAGE (Invitrogen, Life Technologies, Carlsbad, CA) and blotted onto nitrocellulose membrane (GE Healthcare, Little Chalfont, UK). Protein detection was performed using phospho-specific antibodies to *γ*H2AX (Ser139) (Upstate Biotechnology, Lake Placid, NY). Antibodies to H2AX and CDK5RAP2 were purchased from Calbiochem and Bethyl Laboratories, respectively. Anti-*β*-Actin antibodies were purchased from Sigma-Aldrich. Peroxidase-conjugated secondary antibodies were used (Santa Cruz Biotechnology Inc., Dallas, TX), and blots were developed using an enhanced chemiluminescence system, ECL Plus (GE Healthcare), followed by exposure on an autoradiographic film.

### Immunocytochemistry

Fibroblasts from skin biopsies (patient SK-1 II.1 and control) were grown on ethanol-sterilized borosilicate glass cover slips (VWR, thickness 1) to 30% confluence. Cells were fixed with fresh 2% paraformaldehyde in PBS (Invitrogen, Life Technologies, Carlsbad, CA) for 20 min, given three 3-min washes in PBS, and permeabilized for 5 min with 0.4% Triton X-100 (Sigma-Aldrich, St. Louis, MO) in PBS. Cells were then given two 3-min washes in PBS and one 5-min wash in wash buffer (PBS containing 0.2% BSA Faction V, 0.05% Tween 20; Sigma). Cells were blocked for 30 min on a 100 mL drop of blocking buffer (PBS containing 2.5% BSA Fraction V, 0.05% Tween 20 and 10% Natural Goat Serum; Vector Laboratories, Peterborough, UK) placed on Nescofilm in a humid chamber, rinsed briefly with wash buffer and incubated for 1.5 h on 100 mL drops of blocking buffer containing primary antibodies to *α*-tubulin (ab11303; Abcam, Cambridge, UK), pericentrin (ab4448; Abcam) and centrin-1 (Santa Cruz), diluted according to the manufacturer’s instructions. After three further 5-min washes in wash buffer, cells were incubated for 30 min in blocking buffer containing 1:400 dilutions of Alexa Fluor 488 anti-rabbit (A11034; Invitrogen) and Alexa Fluor 568 anti-mouse (A11004; Invitrogen) secondary antibodies. Next, cells were given two 5-min washes in wash buffer and one 5-min wash in PBS and were then mounted in Vectorshield (Vector Laboratories) containing 0.5 mg/mL DAPI (D9542; Sigma-Aldrich). Cells were analyzed and confocal images acquired using a Leica SP1 confocal microscope (Leica, Wetzlar, Germany) using a 100× objective. For quantification, 85 mitoses in Seckel fibroblasts were manually compared with 171 mitoses in wild-type fibroblasts.

### Accession number

*CDK5RAP2*: NM_018249.5

## Results

### Clinical characteristics of index families

**Figure 1:**
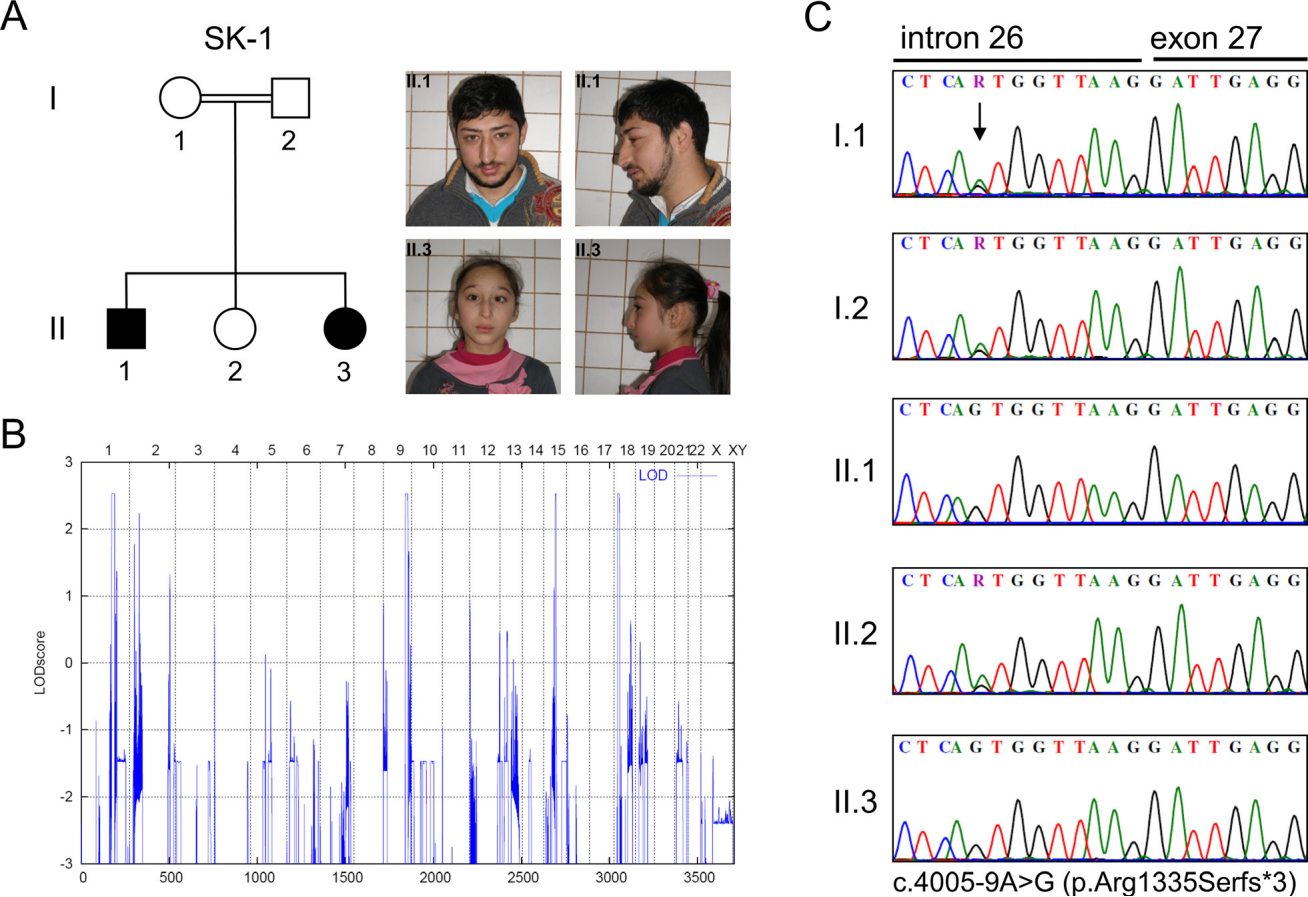
Clinical and molecular findings in the Turkish family SK-1 with Seckel syndrome. (A) Pedigree, front and side views of affected individuals II.1 and II.3 at ages 19 and 10 years, respectively, showing the typical sloping forehead, beaked nose with pointed tip, midface hypoplasia, and microretrognathia. (B) Parametric linkage analysis of the family SK-1 with 20,044 selected SNP markers from the Affymetrix Sty array. LOD scores calculated with ALLEGRO are given along the *y* axis relative to genomic position in cM (centi Morgan) on the *x* axis. The highest scores were obtained for markers on chromosomes 1, 9, 15 and 18. (C) Electropherograms of the identified homozygous *CDK5RAP2* mutation (II.1 and II.3) compared with heterozygous carrier sequences (I.1, I.2 and II.2).

We have clinically assessed two consanguineous families of Turkish and Pakistani ancestry with two individuals in each family diagnosed with a mild form of Seckel syndrome. The index patient of the Turkish family SK-1 (II.1 in Fig.1A) is the first child born to healthy second degree cousins. He was born at term by uncomplicated spontaneous delivery. His birth weight was 2200 g, the birth length and head circumference were not documented. He walked at 13 months of age and spoke his first words at 12 months. He was referred to our clinic at 9 years of age where he was noted to have microcephaly (−4 SD) with a sloping forehead, beaked nose, and midface hypoplasia. Additionally, he had short stature (−3.2 SD) accompanied by skeletal findings such as delayed bone age, fifth finger clinodactyly, and absence of the twelfth ribs. Cognitive impairment was mild-to-moderate (Table1). The patient’s youngest sister (II.3 in Fig.1A), the third child of this family, also presented with Seckel syndrome. She had microcephaly and hip dislocation as a newborn. A cranial MRI from that time did not show any severe structural abnormalities. Achievement of motor milestones was within the normal range, but a mild cognitive impairment became obvious at school age. When we saw her at age 10, she had microcephaly (−4 SD), dysmorphic facial features very similar to those seen in her brother, and short stature (−3.4 SD) (Fig.1A, Table1).

**Table 1:** Comparison of clinical findings in patients with *CDK5RAP2* and digenic mutations described in this study

	SK-1 II.1	SK-1 II.3	SK-2 IV.2	Patient K1600
Sex (no. of patients)	Male	Female	Female	Male
Mutation 1	*CDK5RAP2:* c.4005-9A>G; p.Arg1335Serfs^*^3	*CDK5RAP2:* c.4005-9A>G; p.Arg1335Serfs^*^3	*CDK5RAP2:* c.383+1G>C; p.(Lys129^*^)	*CDK5RAP2:* c.4187T>C; p.(Met1396Thr)
Mutation 2 (digenic mutations)	–	–	–	*CEP152*: c.3014_3015delAAinsT; p.(Lys1005Ilefs^*^16)
Growth				
Head circumference	−4 SD at 9 years	−4 SD at 10 years	−6.2 SD at 2.5 years	−12 SD at 7 years
Length and height	−3.2 SD at 9 years	−3.4 SD at 10 years	−5 SD at 2.5 years	−8.6 SD at 7 years
Facial dysmorphism				
Facial asymmetry	−	−	−	−
Sloping forehead	+	+	+	+
Beaked nose	+	+	+	+
Micrognathia	+	+	+	+
Midface hypoplasia	+	+	+	−
Upslanting palp. fissures	−	+	+	−
Downslanting palp. fissures	−	−	−	+
Strabismus	−	−	+	+
Blepharophimosis	−	−	−	+
Microphthalmia	−	−	−	+
Low-set/malformed ears	−	−	−	+
High-arched palate	+	+	+	+
Selective tooth agenesis	+	−	−	Delayed secondary dentition
Skeletal findings				
Fifth finger clinodactyly	+	+/−	+	+
Brachymesophalangy	−	−	−	+
11 pairs of ribs	+	−	n.a.	n.a.
Delayed bone age	+	+	+	+
Scoliosis	−	−	−	n.a.
Pes planus	−	−	−	n.a.
Other findings				
Intellectual disability	+	+	+	+
Sensorineural hearing loss				
Café au lait spots	+	+	+	−
Other anomalies	−	−	−	Ventralized anus

SD, standard deviation; n.a., not applicable/unknown; palp., palpebral. Accession number *CDK5RAP2*: NM_018249.5.

**Figure 2:**
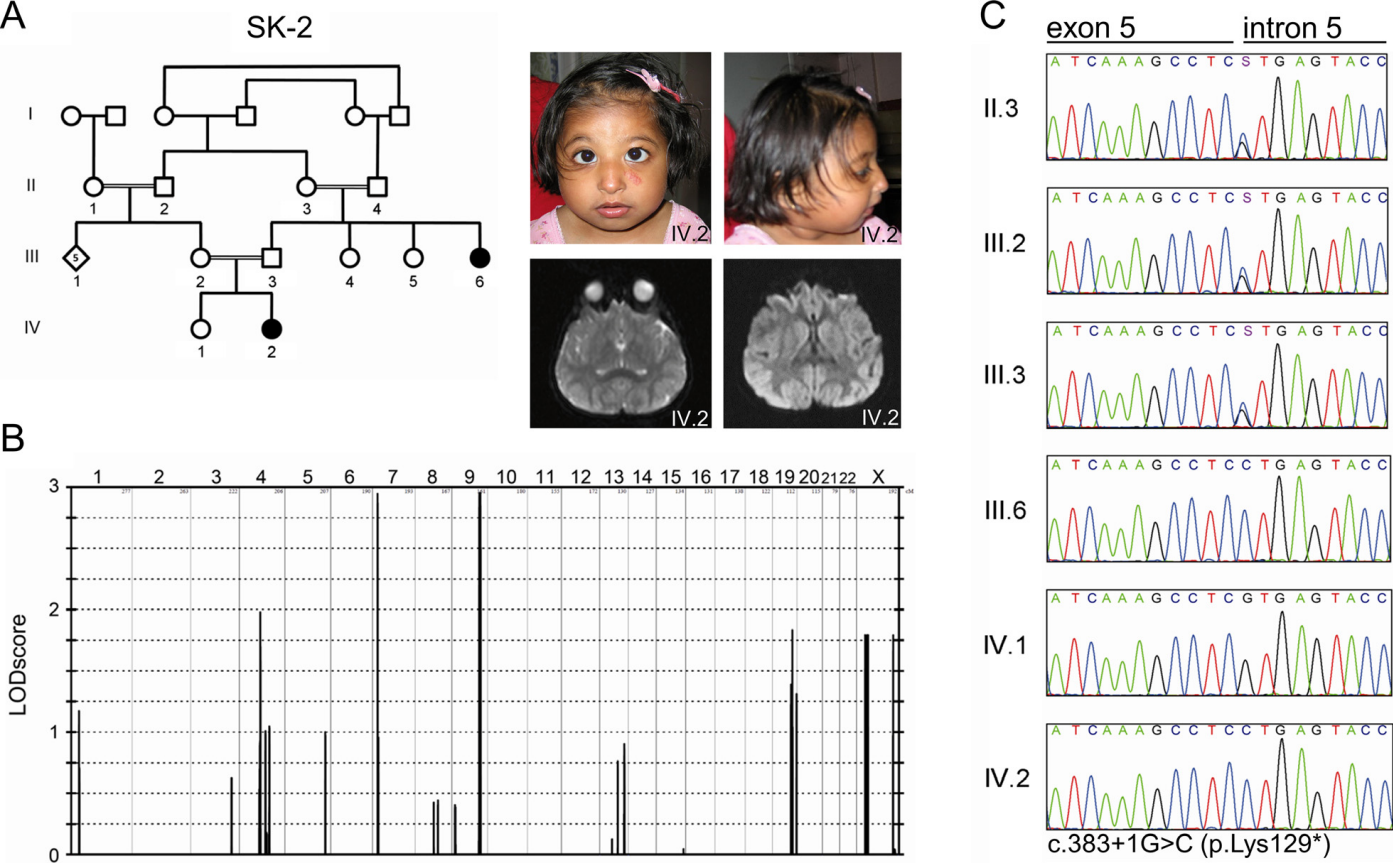
Clinical and molecular findings in family SK-2 with Seckel syndrome. (A) Pedigree of the consanguineous family from Pakistan, front and lateral photographs and MRI images of the affected individual IV.2 at the age of 3 years. (B) Parametric linkage analysis of family SK-2. LOD scores were calculated with ALLEGRO, and the highest scores were obtained for markers on chromosomes 7 and 9. (C) Electropherograms of the identified homozygous *CDK5RAP2* mutation (III.6 and IV.2) compared with heterozygous carrier (II.3, III.2, and III.3) and wild-type sequences (IV.1).

The index patient of the Pakistani family SK-2 (IV.2 in Fig.2A) is the second child of healthy first degree cousins. She was born at term via spontaneous delivery after an uneventful pregnancy. Her birth weight was 2160 g. When she was clinically investigated at 25 months of age, her parents had no concerns about her development. She was microcephalic (−6.2 SD) and had short stature (−5 SD). In addition to her typical facial appearance, her skeletal survey showed signs consistent with the clinical diagnosis of Seckel syndrome, such as mild bowing of the radius and an unusual slope to the radial head, pseudo-epiphyses of the second metacarpals and a mild chevron deformity of the lower end of the femora. Her motor skills were age-appropriate but her attention and language skills were delayed. MRI of the brain at 32 months was normal. The family pedigree showed multiple loops of consanguinity and one paternal aunt was reported to have short stature and to show pointed teeth and child-like behavior. She was not clinically investigated. No mutation in *PCNT* was found in the index patient. She carried a heterozygous SNP in *PCNT*, excluding homozygosity for this gene and thus making a diagnosis of MOPD II unlikely in this family.

### Homozygosity mapping and identification of *CDK5RAP2* as a new Seckel gene

We genotyped DNA samples from all five family members of the Turkish family SK-1 by using the Affymetrix GeneChip® Human Mapping 250K Sty Array as described earlier (Kalay et al. 2011) and obtained four putative loci on chromosomes 1, 9, 15, and 18 with a parametric LOD score of 2.5 each. The shared critical intervals were 21 Mb (chromosome 1, including 202 annotated genes), 11 Mb (chromosome 9, 182 genes), 3.4 Mb (chromosome 15, 62 genes), and 3.2 Mb (chromosome 18, 22 genes) in size (Fig.1B). Genotyping of DNA samples from 6 family members of the Pakistani family SK-2 revealed two putative loci on chromosomes 7 and 9, each with a parametric LOD score of 3. The shared critical intervals were 0.7 Mb (chromosome 7, including 2 annotated genes) and 7.2 Mb (chromosome 9, 57 annotated genes) in size (Fig.2B). Superimposing the linked regions for both families reduced the range of candidate loci to chromosome 9 between SNPs rs10491524 and rs12349082, defining a shared homozygous interval of 3.7 Mb harboring 27 genes.

In a previous study, we reported that mutations in *CEP152*, a protein that contains several coiled-coil domains and is involved in various centrosomal processes, cause Seckel syndrome (Kalay et al. 2011). The similar function of the *cyclin-dependent kinase 5 regulatory subunit-associated protein 2* gene (CDK5RAP2, also named CEP215), located within the shared homozygous region on chromosome 9q33, and the description of recessive mutations in patients with primary microcephaly (Bond et al. 2005) prompted us to consider this gene as a highly relevant candidate.

Sanger sequencing of all 38 coding exons of *CDK5RAP2* in the affected individuals of both families revealed two different homozygous mutations which cosegregated with the disease in either family and were neither found in 150 healthy Turkish and 282 healthy Pakistani control individuals, nor annotated in dbSNP132, the 1,000 Genomes Database, the ∼13,000 alleles of the Exome Variant Server (EVS, National Heart, Lung, and Blood Institute, Exome Sequencing Project, Seattle, WA) or the ∼120,000 alleles of the Exome Aggregation Server (Exome Aggregation Consortium [ExAC], Cambridge, MA). In family SK-1, we identified a homozygous mutation in intron 26 (c.4005-9A>G) (Fig.1C). In family SK-2, we found a homozygous mutation at the donor splice-site of intron 5 (c.383+1G>C) (Fig.2C).

### Mutations in *CDK5RAP2* and their effect on splicing

The c.383+1G>C mutation in family SK-2 is located at the invariant +1 position of the donor splice-site of intron 5. Mutations at this position invariably lead to a loss of the donor splice-site recognition resulting in severe functional effects such as whole-exon skipping, inclusion of intronic sequences or usage of alternative exonic or intronic donor splice sites. The mutation is predicted to alter the CDK5RAP2 transcript composition and – if whole-exon skipping occurs – to create an early stop codon effecting premature protein truncation (p.Lys129*) (Fig.2C).

**Figure 3:**
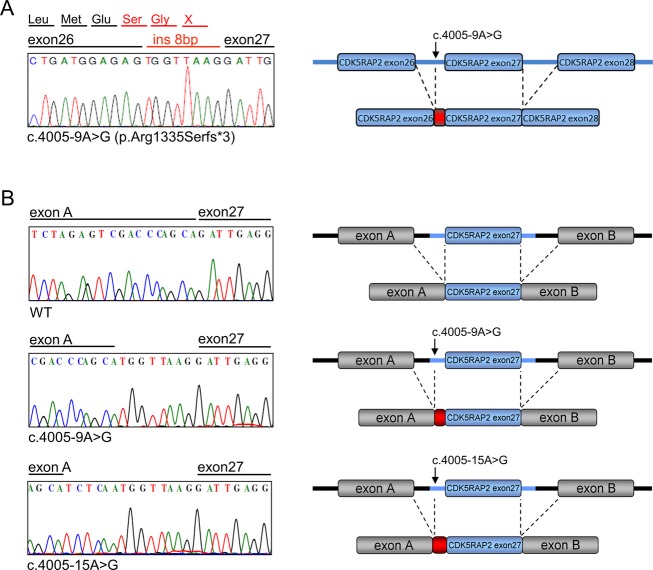
Transcriptional consequences of identified c.4005-9A>G mutation in *CDK5RAP2*. (A) Left: Electropherogram of cDNA derived from the *CDK5RAP2* transcript from patient II.1 of family SK-1 carrying the homozygous *CDK5RAP2* c.4005-9A>G mutation shows alternative splicing of exon 27 leading to an 8 bp insertion and thereby to a frame-shift and a premature stop codon. Right: Schematic representation of the alternatively spliced transcript. (B) Results from the exon-trapping assay. Left: Electropherograms of cDNA-PCR products generated from the wild-type and mutant constructs: The c.4005-9A>G mutation leads to a complete loss of the original splice-site and to insertion of 8 bp. The previously described c.4005-15A>G mutation has a similar effect leading to insertion of 14 bp. Right: Schematic representation of the constructs used for the assay and of the observed alternatively spliced transcripts. Exon A and exon B represent artificial exons of the pSPL3 splicing vector.

To confirm the functional consequence of the identified c.4005-9A>G mutation, we analyzed the cDNA of the index patient of family SK-1 (II.1, Fig.1A), and we showed that this mutation creates a new, stronger acceptor splice-site which completely abolishes the use of the original acceptor site. As predicted, this leads to the insertion of 8 bp, causing a frame-shift and premature truncation of the protein (p.Arg1335Serfs*3, Fig.3A).

The c.4005-15A>G mutation in *CDK5RAP2* was previously described in patients with primary microcephaly (Bond et al. 2005). It affects the same acceptor splice-site as the c.4005-9A>G mutation found in family SK-1. We compared the functional effects of these mutations by employing an exon-trapping experiment. Genomic DNA from the index patient and a control individual containing exon 27 of *CDK5RAP2* and intronic flanking sequences of 600 bp upstream and 700 bp downstream of exon 27 was cloned into the minigene splicing vector pSPL3 and analyzed for functional consequences. Via site-directed mutagenesis we introduced the c.4005-15A>G mutation. We observed that both mutations created a new and strong acceptor splice-site leading to complete loss of the endogenous splice-site (Fig.3B). Thus, we did not detect any significant functional difference between the two mutations in our in vitro system, however, this does not rule out that they may have different functional consequences in vivo.

### CDK5RAP2 protein instability in primary patient fibroblasts

**Figure 4:**
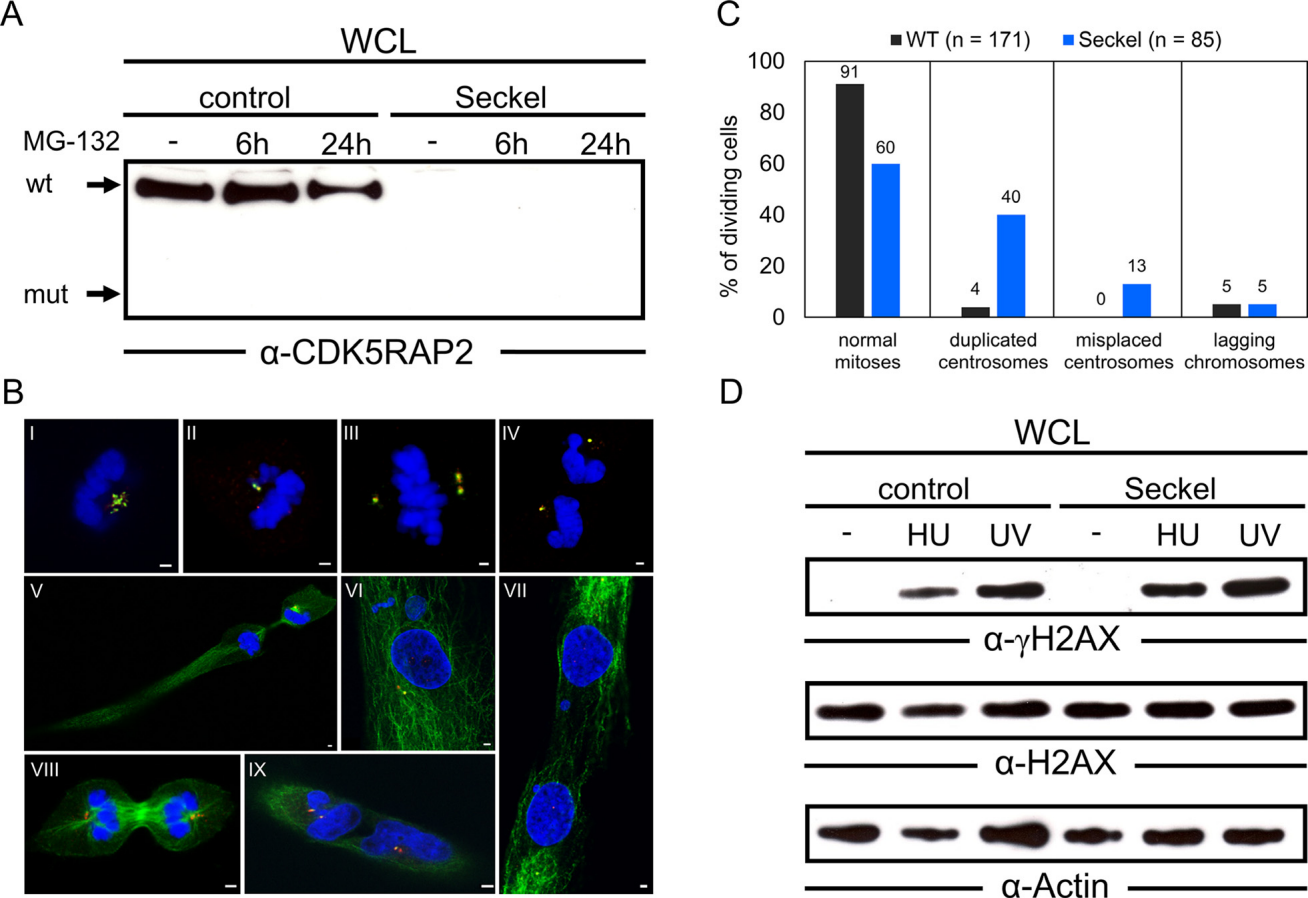
Characterization of CDK5RAP2-deficient Seckel fibroblasts. (A) Western blot (Wb) analysis of CDK5RAP2 expression in wild-type (control) and CDK5RAP2-deficient fibroblasts carrying the *CDK5RAP2* c.4005-9A>G mutation (Seckel): Control and Seckel fibroblasts were treated with MG-132 for the indicated time or left untreated as control. The arrows indicate the position and molecular weight of wild-type CDK5RAP2 (wt) and the expected position and molecular weight of truncated CDK5RAP2 (mut). Wb analysis demonstrates the complete loss of CDK5RAP2 protein in Seckel fibroblasts. (B) Interphase and mitotic morphology of Seckel fibroblasts: Immunofluorescence staining of Seckel fibroblasts with antibodies against centrin (green) and pericentrin (red), and DAPI staining of DNA (blue) (pictures I to IV), showing fragmented centrosomes (I), more centrin than pericentrin (II), multiple centrosomes (III), and misplaced centrosomes (IV). Immunofluorescence staining of Seckel fibroblasts with antibodies against alpha-tubulin (green) and pericentrin (red), and DAPI staining of DNA (blue) (pictures V to IX), showing unevenly distributed cytoplasm (V), micronuclei in addition to a main nucleus (VI), failed separation of daughter cells (VII, IX), and multiple centrosomes during cell division (VIII). Scale bar, 2 *μ*m. (C) Quantification of mitotic and centrosomal anomalies in Seckel fibroblasts. Seckel fibroblasts show abnormalities in 40% of mitoses overall compared to 9% in wt cells. The major abnormal phenotypes observed were duplicated centrosomes and misplaced centrosomes. Values are not additive. (D) DNA damage response in control and Seckel fibroblasts: Wb analysis of HU- and UV-induced phosphorylation of H2AX (Ser139) indicates that the response to DNA-damaging agents is not appreciably altered in CDK5RAP2-deficient fibroblasts.

To investigate whether the identified c.4005-9A>G *CDK5RAP2* mutation causes a complete loss of protein function, we analyzed the expression of CDK5RAP2 in primary fibroblasts established from the index patient of family SK-1 (II.1 in Fig.1A) and from an age and sex-matched healthy control individual. We confirmed the presence of alternatively spliced *CDK5RAP2* mRNA in cells of the affected individual by RT-PCR (Fig.3A). In contrast, no protein expression of truncated CDK5RAP2 could be detected in Seckel fibroblasts, implying a loss of protein function (Fig.4A). Upon treatment of cells with MG-132, a potent inhibitor of the ubiquitin-dependent proteasome system, no residual mutant protein was detected, suggesting that alternative pathways are responsible for the degradation of the truncated protein.

### Centrosomal and mitotic abnormalities in CDK5RAP2-deficient primary fibroblasts

CDK5RAP2 has been shown to be mainly located within centrosomes during all phases of the cell cycle, where it is required for centrosomal cohesion and attachment of centrosomes to the mitotic spindle apparatus (Graser et al. 2007; Barr et al. 2010). Consequently, we analyzed the functional effect of CDK5RAP2 deficiency on centrosomal organization and cellular morphology during interphase and mitosis. We observed severe alterations of centrosomal structures including fragmented centrosomes, unbalanced amounts of centrin and pericentrin, and multiple or misplaced centrosomes (Fig.4B, I–IV). Additionally, we detected significant changes in cell morphology during interphase. Abnormal Seckel cells showed unevenly distributed cytoplasm, micronuclei in addition to a main nucleus, failed separation of daughter cells, and multiple centrosomes during cell division (Fig.4B, V–IX). Quantification of defective mitoses revealed that CDK5RAP2 deficiency leads to an increased proportion of mitotic cells showing centrosomal defects (40% vs. 9% in wild-type cells), i.e., duplicated and misplaced centrosomes (Fig.4C).

As it has been shown that cells from patients with Seckel syndrome lacking either ATR or CEP152 can exhibit abnormal response to DNA-damaging agents such as UV light or HU treatment (Alderton et al. 2004; Griffith et al. 2008; Kalay et al. 2011), we were interested to find out whether CDK5RAP2-deficient Seckel fibroblasts would show similar defects. Therefore, we treated patient fibroblasts from family SK-1 with either HU or UV but did not observe any significant alteration in phosphorylation or activation of H2AX. Thus, CDK5RAP2 deficiency has no obvious impact on ATM/ATR-dependent DNA damage response in this cellular system (Fig.4D).

### Possible digenic inheritance in Seckel syndrome

We continued our molecular analysis of *CDK5RAP2* in 10 additional patients with the clinical diagnosis of Seckel syndrome. In one German patient (K1600), we found a heterozygous *CDK5RAP2* mutation. The patient was the first child born to healthy nonconsanguineous parents. The pregnancy was complicated by intrauterine growth retardation in combination with microcephaly. He was born at term with a birth weight of 1380 g (−8 SD), birth length of 40 cm (−7.25 SD), and OFC of 26 cm (−5.9 SD). The patient showed global developmental retardation, and cranial MRI revealed multiple brain malformations (i.e., partial agenesis of the corpus callosum, holoprosencephaly and partial agenesis of the left hemisphere). The typical facial features of Seckel syndrome; i.e., the sloping forehead, beaked nose with high nasal root, blepharophimosis, strabismus and downslanting palpebral fissures, were quite pronounced in this patient. Additionally, he presented with mild skeletal abnormalities, such as clinodactyly of the second and fifth fingers and ankylosis of the elbows (Table1). Unfortunately, we did not receive permission for publication of photographs.

**Figure 5:**
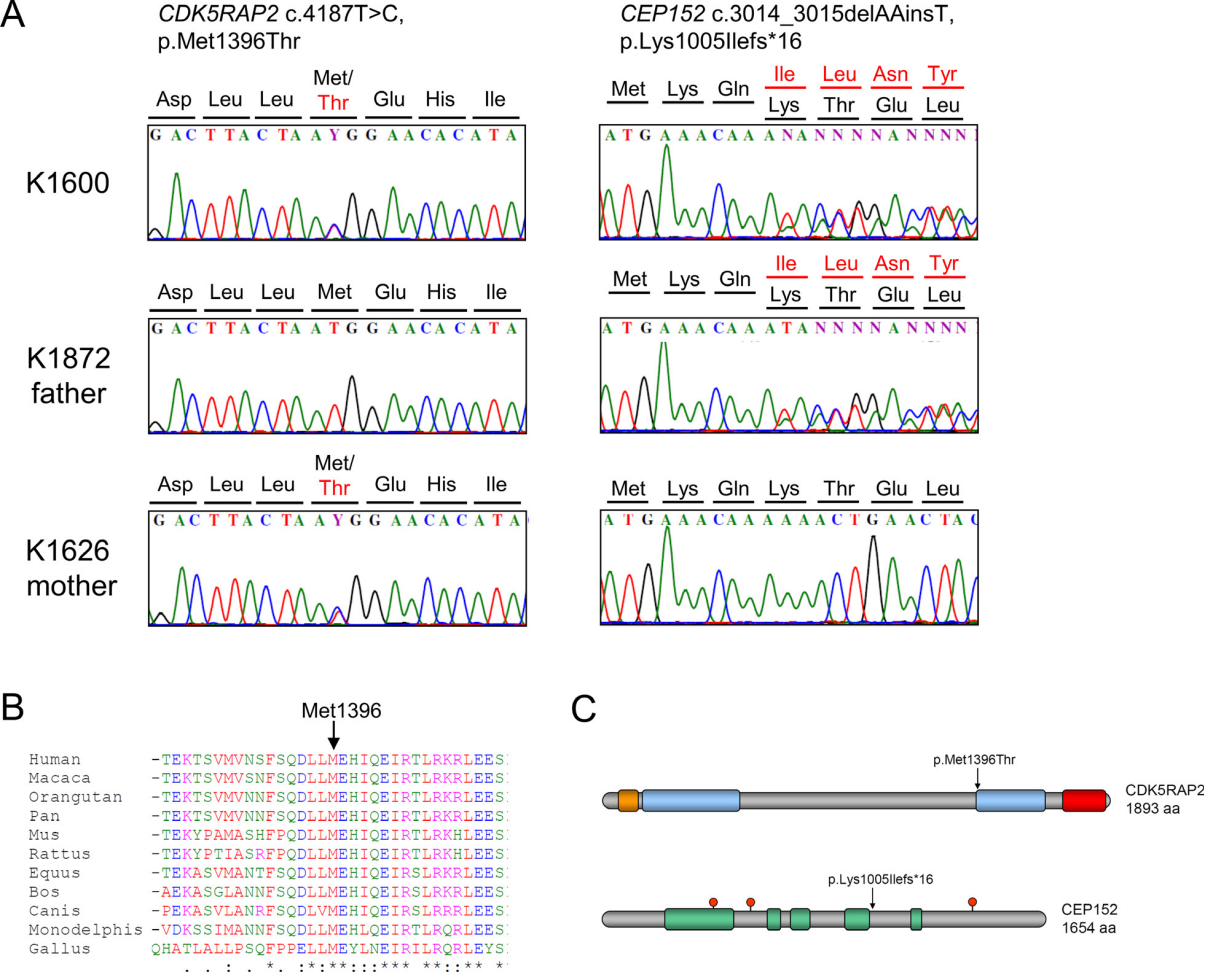
Molecular findings in patient K1600 with Seckel syndrome. (A) Left: Electropherogram of identified heterozygous *CDK5RAP2* mutation compared with heterozygous carrier sequence of the mother (K1626) and wild-type sequence of the father (K1872). Right: Electropherogram of identified heterozygous *CEP152* mutation compared with heterozygous carrier sequence of the father (K1872) and wild-type sequence of the mother (K1626). (B) Amino acid sequence alignment of CDK5RAP2 proteins of different species shows the highly conserved methionine at position 1396. (C) Schematic view of CDK5RAP2 and CEP152 protein domains. Upper picture: Protein structure of CDK5RAP2 with structural maintenance-of-chromosomes (SMC) domains (blue), *γ*-tubulin ring complex-binding domain (orange), and pericentrin interaction domain (red). Lower picture: Protein structure of CEP152 with predicted coiled-coil domains (green) and Thr/Ser-phosphorylation sites (red). The positions of the mutations are marked by arrows.

The heterozygous missense mutation c.4187T>C in *CDK5RAP2* was inherited from the patient’s mother (K1626) and is predicted to replace a highly conserved methionine residue (ConSeq score of 6) within one of the conserved SMC domains of CDK5RAP2 with threonine (Fig.5A–C) (Kraemer et al. 2011). The p.Met1396Thr mutation is predicted to be pathogenic by PolyPhen and was neither found in 150 healthy control individuals, nor in the 1,000 Genomes Database, the Exome Variant Server, or the ExAC browser. As we could not detect a second mutation in *CDK5RAP2* in this patient, we sequenced *ATR* (47 exons), *CEP152* (27 exons), *CENPJ* (17 exons), *CEP63* (14 exons), and *PCNT* (47 exons) for additional mutations. Interestingly, we found the heterozygous small indel c.3014_3015delAAinsT in *CEP152*, inherited from the patient′s father (K1872). The mutation is predicted to cause a frame-shift and a premature stop of translation at amino acid position 1020 (p.Lys1005Ilefs*16), leading to the loss of more than the last third of the CEP152 protein. To exclude any additional larger structural alterations or intronic mutations within *CEP152* that might have been acquired from the mother in terms of compound heterozygous inheritance, we verified the presence of two completely functional *CEP152* mRNA transcripts on cDNA derived from the patient′s mother (data not shown). We could not obtain fibroblasts from this patient for functional studies in order to prove the postulated additive genetic effect of mutations in *CEP152* and *CDK5RAP2* and did not receive consent for whole-exome sequencing.

## Discussion

In this study, we present genetic and functional evidence that biallelic mutations in *CDK5RAP2* cause Seckel syndrome. *CDK5RAP2* encodes the centrosomal protein CDK5RAP2, which was originally identified in a yeast-two-hybrid screen of rat brain libraries to interact with the neuronal Cdk5 activator CDK5R1 (Ching et al. 2000). The human CDK5RAP2 protein consists of 1,893 amino acids and contains several coiled-coil domains as well as two structural maintenance-of-chromosomes (SMC) domains that are known to be important for chromatid cohesion and DNA recombination during mitosis (25 and references therein). Direct protein interaction with CDK5R1 and also pericentrin is mainly effected via the C-terminal region, whereas binding to the *γ*-tubulin ring complex (*γ*-TuRC) and thereby its recruitment to centrosomes occurs via the N-terminal region (Fong et al. 2008; Wang et al. 2010). All genes involved in Seckel syndrome, such as *CEP152*, encode proteins with important functions in mitosis, especially centriole assembly and spindle formation. CDK5RAP2 ties into this family of proteins having been shown to play an important role in centriole cohesion, microtubule dynamics, and spindle orientation (Fong et al. 2009; Barrera et al. 2010). CDK5RAP2 plays an important role during brain development: High levels of expression are observed in regions of rapid cell proliferation, particularly in the neuroepithelium, and CDK5RAP2 has been shown to regulate chromosome segregation in neural progenitors (Nagase et al. 2000; Barr et al. 2010; Lizarraga et al. 2010; Issa et al. 2012). Mice lacking functional CDK5RAP2 exhibit microcephaly, resulting from proliferative and survival defects in neural progenitor cells (Lizarraga et al. 2010). We show that loss of functional CDK5RAP2 in human fibroblasts leads to severe mitotic defects including fragmented centrosomes, unbalanced amounts of centrin and pericentrin, and multiple or misplaced centrosomes in addition to morphologic changes of the dividing cell such as unevenly distributed cytoplasm, micronuclei, multiple centrosomes, and failed separation of daughter cells. A shift in the balance between symmetric and asymmetric cell division and, in consequence, the balance of self-renewal versus differentiation in stem and progenitor cells during neurogenesis heavily impacts embryonic brain development (Fish et al. 2006; Zhong and Chia 2008), and mutations in several genes involved in mitosis have been shown to cause primary microcephaly (Bond and Woods 2006). Recently, Lancaster et al. (2013) could show that cerebral organoids derived from patient-specific iPS cells lacking CDK5RAP2 show premature neural differentiation in progenitor zones at the expense of the neural progenitor pool, resulting in a smaller organoid and thus recapitulating human microcephaly. Moreover, they demonstrated that patient organoid cells displayed aberrantly oriented spindles impeding symmetric expansion of neural stem cells (Lancaster et al. 2013). This result is in line with our observation of misplaced centrosomes and uneven cell divisions in patient fibroblasts. Hence, we propose that increased asymmetric cell division as a consequence of the mitotic errors caused by CDK5RAP2 deficiency may underlie the neurologic phenotype of our patients.

Biallelic mutations in *CDK5RAP2* have been mainly described in patients with primary microcephaly without short stature or skeletal abnormalities (Barr et al. 2010; Pagnamenta et al. 2012; Issa et al. 2013; Tan et al. 2014). The previously described patients with *CDK5RAP2* mutations had head circumferences of –4 to –13 SD. Thus, the degrees of microcephaly are comparable in the previously described and the present patients. Microcephaly was reported to be progressive during childhood in some cases. Most published patients with *CDK5RAP2* mutations had a sloping forehead but did not show other features suggestive of Seckel syndrome (Table2). Sensorineural hearing loss was frequently observed among patients with *CDK5RAP2* mutations but was absent in the present families. The patients presented here have the typical Seckel syndrome phenotype, characterized by the combination of distinct facial features, microcephaly, mental retardation, short stature and skeletal malformations. Patients with Seckel syndrome show mild to severe microcephaly of −4 to −14 SD and short stature of −5 to −11 SD according to Majewski et al. (1982b). Thus the patients from families SK-1 and SK-2 are more at the mild end of the variable Seckel phenotype spectrum, showing head circumferences of −4 to −6 SD and body heights of −3 to −5 SD (Table2).

**Table 2:** Comparison of clinical findings in patients with *CDK5RAP2* mutations described in the literature

	Bond et al. (2005) Family 1*	Bond et al. (2005) Family 2**	Hassan et al. (2007)	Pagnamenta et al. (2012)	Issa et al. (2013)	Tan et al. (2014)	Lancaster et al. (2013)
Sex (no. of patients)	Female (3), male (1)	Female (2)	Female (2), male (2)		Male (2 patients)	Female (1)	
Mutation 1	c.246T>A; p.(Tyr82^*^)	c.4005-15; p.Arg1335Serfs^*^5	c.246T>A; p.(Tyr82^*^)	c.700G>T; p.(Glu234^*^)	c.4441C > T; p.(Arg1481^*^)	c.524_528del; p.(Gln175Argfs^*^42)	c.4546G>T; p.(Glu1516^*^)
Mutation 2 (compound heterozygous)	–	–	–	–	*–*	c.4005-1G>A	c.4672C>T; p.(Arg1558^*^)
Growth							
Head circumference	−6 to −8 SD	P1: −7 SD at 11 years	−4 to −7 SD at 18–30 years	−3.7 SD at birth, −8.9 SD at 6 years	P1: −6.4 SD at 5 years	−8.9 SD at 6 years	−13.2 SD at 3.7 years
		P2: −5 SD at 4 years			P2: −3.5 SD at birth		
Length and height	Normal	Normal	Normal	Normal	Normal	Normal	−6.7 at 3.7 years
Facial dysmorphism							
Facial asymmetry	−	n.a.	−	−	−	−	−
Sloping forehead	+	n.a.	+	+	+	−	−
Beaked nose	+	n.a.	+	−	−	−	−
Micrognathia	−	n.a.	−	−	−	−	−
Midface hypoplasia	n.a.	n.a.	−	−	−	−	−
Abnormal palp. fissures	−	n.a.	−	−	−	−	−
Strabismus	−	n.a.	−	−	−	−	−
Blepharophimosis	−	n.a.	−	−	−	−	−
Microphthalmia	−	n.a.	−	−	−	−	−
Low-set/malformed ears	−	n.a.	−	+	+	−	−
High-arched palate	n.a.	n.a.	−	−	+	−	−
Selective tooth agenesis	n.a.	n.a.	−	−	−	−	−
Skeletal findings	−	n.a.	−	−	−	−	−
Other findings							
Intellectual disability	+	+	+	−	+	+	DD
Sensorineural hearing loss	+	−	−	+	+	−	−
Café au lait spots	−	n.a.	−	−	+	−	−
Other anomalies	Seizures, 1 individual developed ALL	Late closing fontanels, low birth weight	−	Severe GER	Simian creases	−	Prominent eyes, wide-spaced teeth, brain malformation

Accession number *CDK5RAP2*: NM_018249.5.

SD, standard deviation; n.a., not applicable/unknown; palp., palpebral; ALL, acute lymphoblastic leukemia; GER, gastroesophageal reflux; DD, developmental delay.

* Clinical details in Moynihan et al. 2000. *Am. J. Hum. Genet*. 66: 724–727.

** Clinical details in Pagnamenta et al. (2012).

Lancaster et al. (2013) recently described a patient with compound heterozygous *CDK5RAP2* mutations causing primary microcephaly and short stature (37, Table2). Together with these findings our data provide evidence that mutations in this gene also cause Seckel syndrome.

Taking into account that functionally similar mutations within identical genes encoding centrosomal proteins cause a clinical spectrum that ranges from primary microcephaly to severe Seckel syndrome, we propose that the variable phenotypic expression is probably depending on yet unknown genetic modifiers and/or the genetic and functional interaction between several centrosomal proteins. So far, no families have been described in which patients with the same *CDK5RAP2* mutation had either primary microcephaly or Seckel syndrome. However, the intrafamilial variability in the severity of microcephaly (differences up to 3 SD, Table2) may argue in this direction. Different functional consequences in vivo that are missed by in vitro assays may explain interfamilial variability.

In this context, it is interesting to note that we may have identified a case of digenic inheritance of Seckel syndrome, pointing towards an additive genetic effect of mutations in *CDK5RAP2* and *CEP152*. CDK5RAP2 and CEP152 are centrosomal proteins important for centrosomal cohesion and integrity (Bond et al. 2005; Kalay et al. 2011). Regarding a putative additive genetic effect, it is of importance that Conduit et al. recently revealed that *asterless* (*asl*) and *centrosomin* (*cnn*), the Drosophila orthologs of CEP152 and CDK5RAP2, directly interact with each other and that asl contributes to cnn function during centrosome maturation by driving its incorporation into centrioles (Conduit et al. 2010). It was reported that, while Cnn incorporation into the pericentriolar material (PCM) is tightly regulated and mainly dependent on its interaction with asl, cnn itself can only regulate centriolar duplication and maturation when it is present in the PCM (Majewski et al. 1982b). Furthermore, detailed analysis of centrosomal structure and organization of centrioles and the PCM revealed a layered organization of proteins localized in the PCM in interphase cells, with CENPJ at the proximal region at the interface between mother centriole and the PCM and with TUBG1 forming the most distal ring of the PCM (Lawo et al. 2012). Interestingly, both CDK5RAP2 and CEP152 are not only present in the PCM, but located in neighboring toroid structures within the PCM, allowing direct protein–protein interaction. An additive effect of mutations in these two proteins possibly results from a disturbed cooperation in the regulation of centrosomal size during cell cycle. Future functional analyses in animal models will be needed to prove these intriguing hypotheses.

In summary, we describe *CDK5RAP2* mutations in patients with Seckel syndrome, provide evidence that CDK5RAP2 function is necessary for centrosomal integrity and proper cell division, and propose the possibility of a digenic inheritance in centrosomal disorders such as Seckel syndrome.

## Web Resources

The URLs for data presented herein are as follows:

ENSEMBL, http://www.ensembl.org

UCSC Genome Browser, http://www.genome.ucsc.edu

Online Mendelian Inheritance in Man (OMIM), http://ncbi.nlm.nih.gov.omim

ClustalW2, http://www.ebi.ac.uk/Tools/clustalw2/index.htm

PolyPhen, http://genetics.bwh.harvard.edu/pph/

ConSeq, http://conseq.tau.ac.il/index_old_ver.html

Exome Variant Server (EVS), http://evs.gs.washington.edu/EVS/

Exome Aggregation Server (ExAC): http://exac.broadinstitute.org/
